# Neutrophil-to-lymphocyte ratio and incident end-stage renal disease in Chinese patients with chronic kidney disease: results from the Chinese Cohort Study of Chronic Kidney Disease (C-STRIDE)

**DOI:** 10.1186/s12967-019-1808-4

**Published:** 2019-03-15

**Authors:** Qiongjing Yuan, Jinwei Wang, Zhangzhe Peng, Qiaoling Zhou, Xiangcheng Xiao, Yanyun Xie, Wei Wang, Ling Huang, Wenbin Tang, Danni Sun, Luxia Zhang, Fang Wang, Ming-Hui Zhao, Lijian Tao, Kevin He, Hui Xu, C-STRIDE study group

**Affiliations:** 1 Department of Nephrology, Xiangya Hospital, Central South University, 87 Xiangya Road, Changsha, 410008 Hunan China; 2 Renal Division, Department of Medicine, Peking University First Hospital, Institute of Nephrology, Peking University, Key Laboratory of Renal Disease, Ministry of Health of China, Key Laboratory of Chronic Kidney Disease Prevention and Treatment (Peking University), Ministry of Education, Beijing, 100034 China; 3 Center for Data Science in Health and Medicine, Peking University, Beijing, China; 4 Peking-Tsinghua Center for Life Sciences, Beijing, China; 5 Department of Biostatistics, School of Public Health, University of Michigan, Ann Arbor, MI USA

**Keywords:** Neutrophil-to-lymphocyte ratio (NLR), Chronic kidney disease (CKD), End stage of renal disease (ESRD)

## Abstract

**Background:**

Chronic kidney disease (CKD) leads to end-stage renal failure and cardiovascular events. An attribute to these progressions is abnormalities in inflammation, which can be evaluated using the neutrophil-to-lymphocyte ratio (NLR). We aimed to investigate the association of NLR with the progression of end stage of renal disease (ESRD), cardiovascular disease (CVD) and all-cause mortality in Chinese patients with stages 1–4 CKD.

**Methods:**

Patients with stages 1–4 CKD (18–74 years of age) were recruited at 39 centers in 28 cities across 22 provinces in China since 2011. A total of 938 patients with complete NLR and other relevant clinical variables were included in the current analysis. Cox regression analysis was used to estimate the association between NLR and the outcomes including ESRD, CVD events or all-cause mortality.

**Results:**

Baseline NLR was related to age, hypertension, serum triglycerides, total serum cholesterol, CVD history, urine albumin to creatinine ratio (ACR), chronic kidney disease-mineral and bone disorder (CKD-MBD), hyperlipidemia rate, diabetes, and estimated glomerular filtration rate (eGFR). The study duration was 4.55 years (IQR 3.52–5.28). Cox regression analysis revealed an association of NLR and the risk of ESRD only in patients with stage 4 CKD. We did not observe any significant associations between abnormal NLR and the risk of either CVD or all-cause mortality in CKD patients in general and CKD patients grouped according to the disease stages in particular.

**Conclusion:**

Our results suggest that NLR is associated with the risk of ESRD in Chinese patients with stage 4 CKD. NLR can be used in risk assessment for ESRD among patients with advanced CKD; this application is appealing considering NLR being a routine test.

*Trial registration* ClinicalTrials.gov Identifier NCT03041987. Registered January 1, 2012. (retrospectively registered) (https://www.clinicaltrials.gov/ct2/show/NCT03041987?term=Chinese+Cohort+Study+of+Chronic+Kidney+Disease+%28C-STRIDE%29&rank=1)

**Electronic supplementary material:**

The online version of this article (10.1186/s12967-019-1808-4) contains supplementary material, which is available to authorized users.

## Background

Chronic kidney disease (CKD) is a growing health problem with an estimated prevalence of 10.8–16% in major developed and developing countries [1, 2]. Individuals with CKD are at risk of progressive kidney failure, cardiovascular events, and death [3, 4].

Nonmicrobial inflammation contributes to CKD progression and fibrosis [5]. The neutrophil count reflects inflammation, while the lymphocyte count indicates the status of general stress and nutrition. The neutrophil-to-lymphocyte ratio (NLR) in CKD patients provides information on the inflammation status [6]. It is suggested that NLR is a complementary prognostic marker for evaluating the cardiovascular risk in CKD3-5 patients [6]. Studies demonstrated that an increase in neutrophil count coupled with a reduction in lymphocyte counts predicts mortality in hemodialysis patients [7] and peritoneal dialysis patient [8]. Also, NLR indicates the rate of stage 4 chronic kidney disease progressing to dialysis [9]. Nevertheless, studies of NLR for its prognosis potential towards ESRD, CVD and all-cause mortality in patients with stage 1–4 CKD other than stage 5 CKD are rare. Since NLR is readily derived from complete blood count tests, its potential as a predictor should be investigated in a large cohort of patients with stage 1–4 CKD.

The objective of this study was to evaluate whether NLR, a simple marker of chronic systemic inflammation, predicts the progression of ESRD, CVD and all-cause mortality among CKD1–4 patients in the Chinese Cohort Study of Chronic Kidney Disease (C-STRIDE), the first national prospective CKD cohort of the Chinese population.

## Materials and methods

The design, methods, and baseline characteristics of the Chinese Cohort Study of Chronic Kidney Disease (C-STRIDE) study population has been published; this is a prospective investigation involving 39 clinical centers in 28 cities of 22 provinces in China [10]. The design of C-STRIDE has been described in detail elsewhere [11]. A total of 3358 participants were included until December 31, 2017 after exclusion of patients with missing value of key demographic variables (including blood routine and blood lipid) or loss of follow-up. Of these 3358 participants, 938 patients have NLR and other relevant clinical variables and were used in the current analysis. The C-STRIDE study was approved by the ethics committee of Peking University First Hospital and was in adherence with the Declaration of Helsinki. All participants have consented this study. During the study visit, all C-STRIDE study data were collected by trained staff according to the study protocol [12]. Data were obtained by questionnaires, anthropometric measurements, collection of blood and urine specimens. The albumin/creatinine ratio (ACR) stage was categorized according to an analysis of a spot urine sample: A1 (normoalbuminuria), ACR < 30 mg/g creatinine; A2 (microalbuminuria), 30 ≤ ACR < 300 mg/g creatinine; or A3 (macroalbuminuria), ACR ≥ 300 mg/g creatinine. GFR was estimated from serum creatinine measurements and demographic characteristics by the Chronic Kidney Disease Epidemiology Collaboration (CKD-EPI) equation [1, 13]. Smoking was defined as currently smoking or had ever smoked. Alcohol consumption was defined as a habitual drinker (drink once a day or more). Hypertension was defined as a systolic blood pressure (BP) ≥ 140 mmHg and/or a diastolic BP ≥ 90 mmHg, or a self-reported history of hypertension. Levels of hemoglobin, NLR, fasting blood glucose, serum triglycerides, total cholesterol, LDL-cholesterol, HDL cholesterol, uric acid, serum phosphate, serum calcium, hs-CRP, iPTH, HbA1c, were documented. Plain lateral abdominal x-ray film showed abdominal aortic calcification. CKD-MBD was defined as a triad of interrelated abnormalities of serum biochemistry (serum phosphate levels > 1.49 mmol/L or < 0.87 mmol/L; serum calcium levels > 2.57 mmol/L or < 2.1 mmol/L, and iPTH > 70 pg/mL or < 35 pg/mL); or calcification of lateral abdominal x-ray film. Patients were considered to have diabetes mellitus if they had a fasting glucose ≥ 7.0 mmol/L; an HbA1c ≥ 6.5%; took insulin or other anti-diabetic medications; or reported a history of diabetes. CVD history was defined as the past occurrence of a myocardial infarction, admittance into a hospital for congestive heart failure, or severe cardiac arrhythmia incidents (resuscitated cardiac arrest, ventricular fibrillation, sustained ventricular tachycardia, paroxysmal ventricular tachycardia, atrial fibrillation or flutter, severe bradycardia, or heart block). Hyperlipidemia was defined as total cholesterol level ≥ 5.7 mmol/L or an LDL-cholesterol level ≥ 3.6 mmol/L. Hyperuricemia was defined as a serum concentration of uric acid ≥ 420 μmol/L for men and ≥ 360 μmol/L for women. The abnormal of hsCRP was ≥ 3 mg/dL [14]. The kidney disease outcomes evaluated in this paper are three facets: (1) ESRD that was defined as progression to hemodialysis, peritoneal dialysis or renal transplantation; (2) CVD risk that was evaluated based on onset of CVD events (myocardial infarction, heart failure, arrhythmia and cerebrovascular disease and peripheral arterial disease) and (3) all-cause mortality (deaths). Doctors at the clinical centers were requested to submit related clinical data to Renal Institute of Peking University. All events were adjudicated by an independent committee consisting of specialists.

### Statistical analyses

Demographic information and other baseline variables were described using mean ± SD or median and interquartile range for continuous variables and frequency and proportion for categorical variables. Chi-square or Fisher’s exact test was used for comparison of categorical variables between groups, while Mann–Whitney U test or unpaired Student’s t test was used for continuous variables. We identified the cutoff point for NLR level using maximally selected log-rank statistics. Prevalence of events according to NLR categorical variables and estimate survival time for each category are calculated with Kaplan–Meier Survival and time-to-event analysis of outcomes were performed using Cox proportional hazards model, including adjustment for potential confounding factors. Covariates for the models were selected based on prior knowledge about the factors that could be potential confounders of the associations of NLR with ESRD. The potential confounders including age (continuous), gender (male vs. female), smoking (yes vs. no), drinking more than once a day (yes vs. no), clinical characteristics [hypertension (yes vs. no), CKD-MBD (yes vs. no), diabetes (yes vs. no), CVD (yes vs. no), hyperlipidaemia (yes vs. no), hyperuricemia (yes vs. no)], hemoglobin (continuous), eGFR (continuous), ACR categories (A1, A2, A3). A *p* value < 0.05 was regarded as statistically significant. Proportional hazards assumptions were verified by testing the interaction with time using the likelihood ratio test, which yielded non-significant *p* values. The results are presented as hazard ratios (HRs) with 95% confidence intervals (CIs). *p* values for trend were given by treating dichotomy NLR as a continuous variable. We hypothesized that the effect of NLR might be modified by the CKD stage. The interaction terms were generated between NLR and each of the eGFR levels (CKD1-2 vs. all others, CKD3 vs. all others, CKD4 vs. all others). A stratified analysis in the prediction of ESRD events was conducted by the stages of CKD. A series of sequential models were fit to evaluate the effect of adding certain sets of covariates. All statistical analyses were performed using SPSS 24.0 for Windows (SPSS Inc., Chicago, IL, USA).

## Results

### Baseline characteristics of the patients

**Fig. 1:**
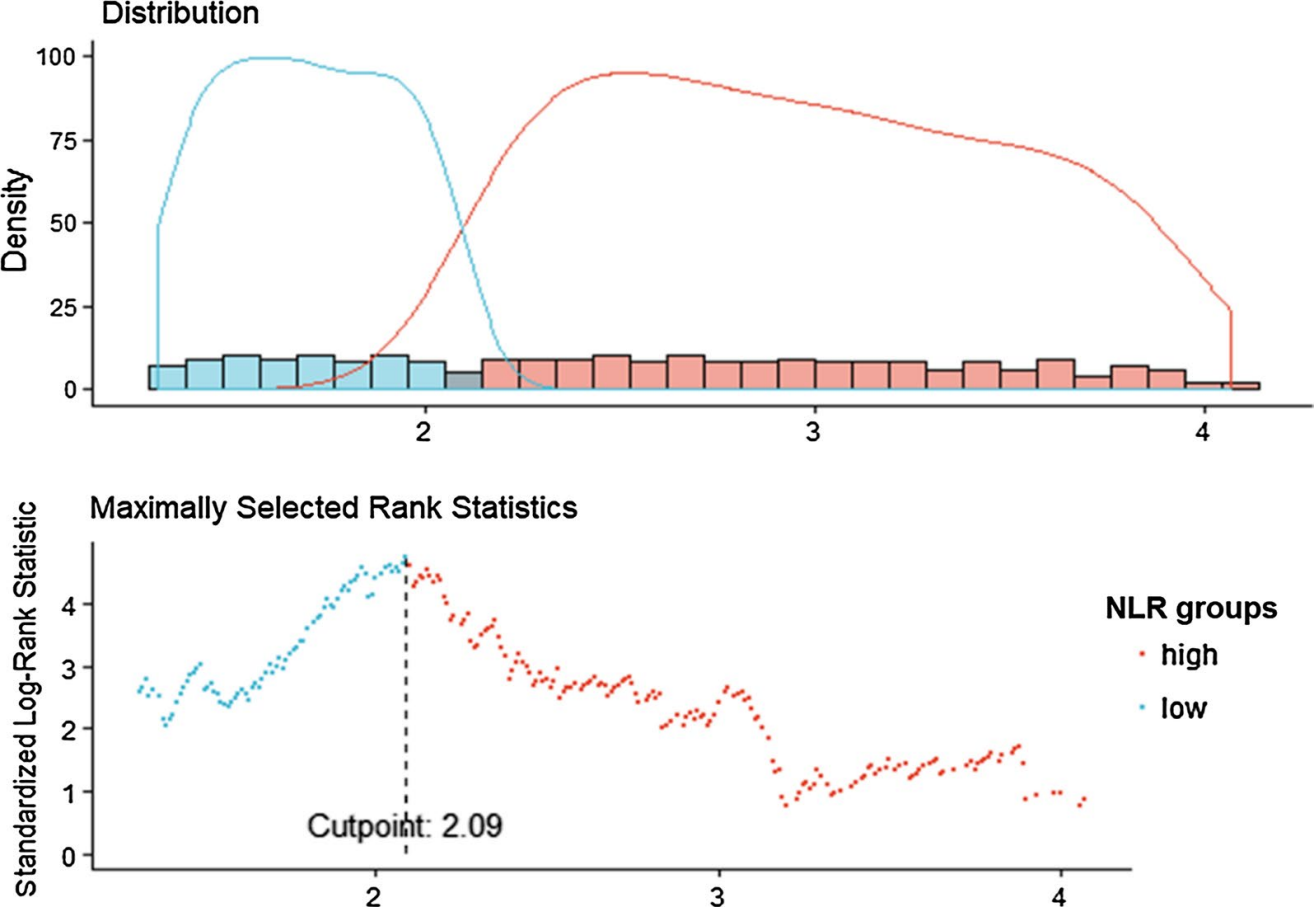
Maximally selected log-rank statistics for cutoff point of NLR

Among the total 938 CKD patients from C-STRIDE, the median follow-up time for the adverse outcomes was 4.55 (IQR 3.52–5.28) years. Baseline characteristics of the study population are described in Table 1. The mean age of the study population was 52.8 years; 58% of patients were male. 34.2% of patients were reported ever smoking and 21% of patients were documented drinking ≥ 1 times per day. At the baseline, 360 (38.4%) patients had an eGFR greater than 60 mL/min 1.73 m^−2^; 345 (36.8%), and 233 (24.8%) patients were in CKD stage 3 and 4, respectively. Baseline demographics and biochemical measurements of CKD patients according to dichotomy of baseline NLR are summarized in Table 1. Higher baseline NLR levels were associated with older age, higher blood pressure, hyperlipidaemia, CVD, CKD-MBD, diabetes, but lower levels of eGFR. The cutoff point for the serum NLR level for renal replacement progression was 2.09 (Fig. 1). We also compare the baseline characteristics of 2420 patients without record of NLR in 3359 participants to those who has the record of NLR (Additional file 1: Table S1). There are differences in ever smoking, BMI category, hypertension, uric acid, serum triglycerides, hyperuricemia, LDL cholesterol, HDL cholesterol, hyperlipidaemia, serum phosphorus (sP), HCO^3−^ and eGFR between NLR group and non-NLR group, but there are no difference in age, sex, drinking, HGB, total serum cholesterol, cardiovascular disease (CVD), ACR, serum calcium (sCa) and diabetes.

**Table 1:** Baseline demographic characteristics of participants of C-STRIDE Study according to NLR

Variable	Total (n = 938)	NLR ≥ 2.09 (n = 520)	NLR < 2.09 (n = 418)	*p* value
Age (year)*	52.8 (14.14)	54.0 (14.48)	51.3 (13.58)	0.047**
Sex (men)^$^	544 (58.00%)	304 (58.46%)	240 (57.42%)	0.747
Ever smoking^$^	311 (34.2%)	180 (35.7%)	131 (32.3%)	0.276
Drinking ≥ 1 times per day^$^	189 (21.00%)	101 (20.2%)	88 (22.0%)	0.510
BMI category (kg/m^2^)^#^	24.61 (22.04–27.34)	24.61 (22.01–27.42)	24.59 (21.99–27.31)	0.944
HGB (g/L)*	129.2 (22.78)	125.7 (23.00)	133.70 (21.71)	0.183
Systolic blood pressure (mmHg)*	127.51 (16.71)	129.91 (17.88)	124.92 (14.96)	0.537
Diastolic blood pressure (mmHg)*	80.13 (10.48)	81.11 (11.01)	79.08 (9.78)	0.918
Antihypertensive medications token in 2 weeks^$^	507 (69.5%)	288 (74.0%)	219 (64.4%)	0.005**
Hypertension^$^	538 (57.4%)	303 (58.3%)	235 (56.2%)	< 0.001**
Uric acid (μmol/L)*	373.59 (132.08)	376.59 (136.42)	369.87 (126.55)	0.208
Serum triglycerides (mmol/L)^#^	1.66 (1.18–2.39)	1.61 (1.13–2.26)	1.79 (1.21–2.59)	0.021**
Hyperuricemia^$^	407 (44.3%)	235 (46.2%)	172 (42.0%)	0.201
Total serum cholesterol (mmol/L)^#^	4.70 (4.01–5.57)	4.59 (3.94–5.39)	4.82 (4.12–5.89)	0.003**
LDL cholesterol (mmol/L)^#^	2.73 (2.22–3.40)	2.72 (2.20–3.36)	2.74 (2.23–3.48)	0.369
HDL cholesterol (mmol/L)^#^	1.12 (0.91–1.34)	1.12 (0.93–1.34)	1.12 (0.89–1.34)	0.729
Hyperlipidaemia^$^	194 (26.6%)	98 (23.7%)	96 (30.5%)	0.039**
Cardiovascular disease^$^	124 (13.40%)	81 (15.80%)	43 (10.40%)	0.017**
ACR (mg/g creatinine)^#^	343.05 (75.60–780.51)	420.50 (112.11–312.90)	290.01 (48.20–666.86)	0.000**
ACR group^$^	814	447	367	0.006**
1 < 30 mg/g	127 (15.6%)	56 (12.5%)	71 (19.3%)	
2 = 30–299 mg/g	248 (30.5%)	130 (29.1%)	118 (32.2%)	
3 ≥ 300 mg/g	439 (53.9%)	261 (58.4%)	178 (48.5%)	
sCa (mmol/L)*	2.22 (0.18)	2.23 (0.18)	2.22 (0.18)	0.735
sP (mmol/L)^#^	1.17 (1.05–1.31)	1.17 (1.04–1.31)	1.18 (1.05–1.32)	0.925
CKD-MBD^$^	467 (54.6%)	274 (58.1%)	193 (50.3%)	0.023**
HCO^3−^ (mmol/L)*	25.70 (3.78)	25.54 (3.99)	25.91 (3.49)	0.060
Diabetes^$^	200 (23.8%)	135 (29.4%)	65 (17.1%)	0.000**
eGFR (mL/min/1.7 m^2^)*	57.22 (32.67)	48.73 (29.63)	67.78 (33.24)	0.000**
eGFR group^$^	938			
≥ 60 mL/min/1.73 m^2^	360 (38.4%)	139 (26.7%)	221 (52.9%)	0.000**
30–60 mL/min/1.73 m^2^	345 (36.8%)	212 (40.8%)	133 (31.8%)	
15–30 mL/min/1.73 m^2^	233 (24.8%)	169 (32.5%)	64 (15.3%)	
High hs-CRP	146 (15.6%)	88 (16.9%)	58 (13.9%)	0.414

The number of missing values for each variable: NLR 0, age 0, eGFR 0, sex 0, education 31, ever smoking 51, drinking ≥ 1 times per day 60, BMI category 211, anemia 0, systolic blood pressure 186, diatolic blood pressure 186, antihypertensive medications token in 2 weeks 334, uric acid 30, total serum cholesterol 338, serum triglycerides 347, LDL cholesterol 428, HDL cholesterol 425, CKD-MBD 0, cardiovascular disease 28, ACR 149, metabolic acidosis 589, pathogenesis chronic 0, high Hs-CRP 668

The denominator of percentage is number of the variable

* The variable is numerical and statistics is mean (standard deviation), p-value calculated based on T test

^#^The variable is numerical and statistics is Median (Interquartile range), p-value calculated based on Wilcoxon test

^$^The variable is character and statistics is frequency (percentage), p-value calculated based on Chi-square test

** Statistically significant at 0.05

### The incidence rates of ESRD other than CVD and all-cause mortality events were associated with NLR

**Fig. 2:**
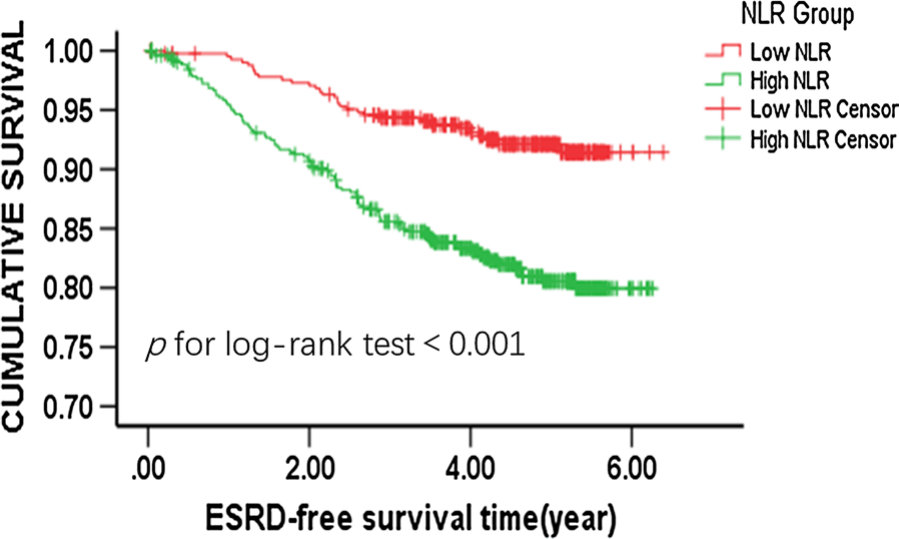
Kaplan–Meier curve for ESRD events according to binary of NLR

The incidence rates of ESRD, CVD and all-cause mortality events according to binary of NLR levels are shown in Table 2. During the median follow-up of 4.55 (IQR 3.52–5.28) years, there were 123 ESRD events occurred. ESRD rates were 3.14 per 100 person-years. Higher incidence rate of ESRD events was observed with increases in NLR (Fig. 2, *p* for log-rank test < 0.001). There were 57 CVD and 43 all-cause mortality events; the occurance of both events was not associated with NLR levels (Table 2). Furthermore, we did not detect any significant correlations among hsCRP, another marker of inflammation, with all-cause mortality rates, ESRD rates and CVD events (Additional file 1: Table S2).

**Table 2:** Relationship between NLR levels and ESRD, CVD and all-cause mortality events rates

NLR binary	Number of events	Events per 100 person-years	*p* for log-rank
ESRD events			
< 2.09 (N = 418)	31 (7.4%)	1.76	< 0.001
≥ 2.09 (N = 520)	92 (17.7%)	4.27	
Total	123 (17.02%)	3.14	
CVD events			
< 2.09 (N = 418)	19 (4.5%)	1.03	0.081
≥ 2.09 (N = 520)	38 (7.3%)	1.69	
Total	57 (6.1%)	1.39	
All-cause mortality events			
< 2.09 (N = 418)	14 (3.4%)	0.75	0.118
≥ 2.09 (N = 520)	29 (5.5%)	1.26	
Total	43 (4.6%)	1.03

### Associations between NLR and ESRD

The associations between NLR and ESRD are shown in Table 3. After adjustment for demographic and traditional ESRD risk factors, as well as the baseline eGFR categories and ACR, baseline NLR was independently associated with the occurrence of ESRD in CKD stage 4 patients, with an HR value 2.12 (95% CI 1.10–4.10) compared with the lower NLR (*p *= 0.025).

**Table 3:** Association of NLR with ESRD events among total CKD patients and stratified by stage of CKD

NLR ratio	Model 1	Model 2
	HR (95% CI)	HR (95% CI)
Total CKD patients (n = 938)		
< 2.09	1.00 (Ref)	1.00 (Ref)
≥ 2.09	2.59 (1.72, 3.90)	1.16 (0.74, 1.81)
p for trend	< 0.001	0.512
eGFR ≥ 60 mL/min (n = 360)		
< 2.09	1.00 (Ref)	1.00 (Ref)
≥ 2.09	1.77 (0.36, 8.87)	1.57 (0.21, 11.98)
*p* for trend	0.485	0.663
30 ≤ eGFR < 60 mL/min (n = 345)		
< 2.09	1.00 (Ref)	1.00 (Ref)
≥ 2.09	0.98 (0.50, 1.92)	0.507 (0.23, 1.11)
*p* for trend	0.95	0.089
15 ≤ eGFR < 30 mL/min (n = 233)		
< 2.09	1.00 (Ref)	1.00 (Ref)
≥ 2.09	2.11 (1.18, 3.76)	2.124 (1.10, 4.10)
*p* for trend	0.012	0.025

Model 1: Adjusted for age, gender

Model 2: Model 1 + smoking, drinking, diabetes, hypertension, cardiovascular diseases, hyperuricemia, urine albumin/creatinine ratio, chronic kidney disease-mineral and bone disorder, hemoglobin, hyperlipidaemia, eGFR

## Discussion

The current study suggests that baseline NLR is associated with an increased risk of ESRD in pre-dialysis patients with stage 4 CKD; the association was independent of traditional risk factors of CKD including ACR and eGFR.

In CKD patients, declines in glomerular filtration rate are associated with increases in the risk of CVD and rapid progression of CKD to end-stage renal disease and mortality [15, 16]. CKD is a chronic inflammatory condition and remains a substantial economic burden on the patient, caregiver and society [17]. Inflammation is one of the most important initiators of progressive tubule-interstitial fibrosis, which usually culminates in ESRD [18]. Several studies have reported that inflammation plays a role in the decline of kidney function [19]. Anti-inflammatory treatment in tubulointerstitial fibrosis of CKD may have renal protective effects [20–23]. An alternative marker of systemic inflammation, c-reactive protein (CRP) has been shown to be a superior prognostic marker if it is persistently elevated [24, 25]. HsCRP was found to be a predictor of mortality and ESRD in pre-dialysis patients with chronic kidney disease [26]. HsCRP, which is synthesized mainly in the liver, only marginally increased even in patients with active infection [24]. Furthermore, it is non-specific and may not fully capture all forms of inflammation [27]. For example, the Multi-Ethnic Study of Atherosclerosis (MESA) demonstrated obesity being independently associated with subclinical atherosclerosis irrespective of hsCRP, with no additive effect when elevated hsCRP was present [28]. What is more, hsCRP is influenced by other factors such as body mass index, weight loss, smoking, active alcohol consumption and diabetes [29]. Similar in our study, we observed that hsCRP was not correlated with the outcome in CKD stage 1–4 patients. hsCRP is thus likely not a good inflammation indicator in CKD patients; other inflammation biomarkers should be investigated to stratify patients with stage 1–4 CKD who are at risk of poor prognosis and to monitor treatment effects.

Studies with relatively small-scale have recently been conducted on whether NLR may be a predictor of CKD progression. The balance between the inflammatory and immune response is also reflected by NLR [7], which shows chronic low-grade inflammation. In addition to a variety of well-known risk factors for CKD progression, this study was aimed to evaluate the relationship between NLR and CKD progression. We used NLR as a surrogate marker of systemic inflammation. NLR is composed of two different complementary immune pathways [30], and is less likely to be influenced by various physiological conditions such as dehydration. Because some inflammatory cytokines, such as hsCRP, interleukin-6 and TNF-α are limited to research for nonconventional tests, NLR would be more easily available for clinical practice [31]. NLR displays a prognostic value for proteinuria [32, 33], mortality and RRT [34]. Nevertheless, recent evidence indicates NLR was not an independent predictor of CKD progression in CKD stage 2–4 patients [35]. In our study of a large chinese CKD population for relationship between NLR, ESRD, CVD, or all-cause mortality, our longitudinal study revealed NLR as an independent risk factor of ESRD only in patients with stage 4 CKD after adjusting for classic risk factors of CKD including ACR and eGFR. These patients have severely damaged renal function prior to dialysis [36]; chronic low-grade inflammation will likely promote disease progression towards ESRD in stage 4 CKD patients. In this regard, NLR may not be an independent predictor of CKD progression in patients with CKD at early stages [35]. In renal replacement patients, including hemodialysis and peritoneal dialysis, evidence suggests that NLR predicts CVD and all-cause mortality [7]. Nevertheless, in our study, this relationship could not be demonstrated in pre-dialysis patients. It remains possible that NLR predicts CVD and all-cause mortality only in stage 5 CKD patients.

Our study has several limitations. First, although most well-established risk factors of CKD progression were included in our multivariable regression models, the possibility of residual confounding still exists. Secondly, despite using a large patient cohort organized by multiple centers, risk factor exposures, CVD risk and deaths in some regions were affected by data shortages. Thirdly, the NLR only captured in 938 of 3358 patients, selection bias could not be excluded. Forthly, the cohort has a relatively short duration of follow-up and a limited number of cardiovascular events and death [37], which limited our power to investigate the association between the levels of NLR and CVD and all-cause mortality.

## Conclusion

NLR may independently predict the risk of ESRD in patients with stage 4 CKD. NLR is easily available, which adds valuable prognostic information to the well-established clinical and biochemical prognostic biomarkers. NLR could be used to improve risk-stratification of patients with stage 4 CKD. Future studies are warranted to see whether stage 4 CKD patients with elevated NLR levels will benefit from anti-inflammatory therapies and interventions.

## Additional file

**Additional file 1: Table S1.** Baseline demographic characteristics of participants of C-STRIDE Study between population with NLR and without NLR. **Table S2.** Relationship between hsCRP and ESRD, CVD and all-cause mortality events rates.

## Abbreviations

CKD: Chronic kidney disease
NLR: neutrophil-to-lymphocyte ratio
ESRD: end stage of renal disease
CVD: cardiovascular disease
ACR: albumin to creatinine ratio
CKD-MBD: chronic kidney disease-mineral and bone disorder
eGFR: estimated glomerular filtration rate
C-STRIDE: the Chinese Cohort Study of Chronic Kidney Disease
CKD-EPI: the Chronic Kidney Disease Epidemiology Collaboration
HRs: hazard ratios
CIs: confidence intervals
sP: serum phosphorus
sCa: serum calcium
MESA: Multi-Ethnic Study of Atherosclerosis
