# Supplementary material for: Neutrophil-to-lymphocyte ratio and incident end-stage renal disease in Chinese patients with chronic kidney disease: results from the Chinese Cohort Study of Chronic Kidney Disease (C-STRIDE)
Source: J Transl Med. 2019 Mar 15;17:86. doi: 10.1186/s12967-019-1808-4 (PMC6420746; doi:10.1186/s12967-019-1808-4)
Supplement: Supplementary file 1 — Additional file 1: Table S1. Baseline demographic characteristics of participants of C-STRIDE Study between population with NLR and without NLR. Table S2. Relationship between hsCRP and ESRD, CVD and all-cause mortality events rates. [file 12967_2019_1808_MOESM1_ESM.rtf]

Table S1 Baseline demographic characteristics of participants of C-STRIDE Study between population with NLR and without NLR

Variable	Total
(n = 3358)	Population with NLR
（¨n=938）©	Population without NLR（¨n=2420）©	P value	
Age (year) *	53.14(13.75)	 52.8(14.14)	 53.27 (13.60)	0.116	
Sex (men) $	 1981（¨59%）©	 544(58.00%)	 1437(59.4%)	0.464	
Ever smoking $	 1226(37.7%)	 311(34.2%)	 915(37.8%)	0.035**	
Drinking≥1 times per day $	 669(20.7%)	 189(21.00%)	 480(20.6%)	0.806	
BMI category (kg/m2) #	 24.31(22.03-26.78)	 24.61(22.04-27.34)	 24.22(22.03-26.64)	0.028**	
HGB(g/L) *	127.73(22.38)	129.25(22.78)	125.79(21.72)	0.323	
Systolic blood pressure (mmHg) *	129.23(17.71)	127.51 (16.71)	129.93(18.06)	0.525	
Diastolic blood pressure (mmHg) *	 80.78(11.55)	 80.13(10.48)	 81.05(11.95)	0.795	
Antihypertensive medications token in two weeks $	1973(72.9%)	 507(69.5%)	 1466(74.1%)	0.019**	
Hypertension$	2083(62.0%)	538(57.4%)	1545(63.8%)	0.002**	
Uric acid (umol/L) *	403.67(121.73)	373.59(132.08)	416.14(114.91)	0.001**	
Serum triglycerides (mmol/L) #	1.80(1.24-2.56)	 1.66(1.18-2.39)	  1.83(1.28-2.62)	<0.001**	
Hyperuricemia$	1662(49.5%)	407(44.3%)	1255(51.9%)	0.000**	
Total serum cholesterol (mmol/L) #	4.77(3.94-5.84)	  4.70(4.01-5.57)	  4.80(3.90-5.96)	0.136	
LDL cholesterol (mmol/L) #	2.62(2.10-3.31)	  2.73(2.22-3.40)	  2.58(2.08-3.28)	<0.001**	
HDL cholesterol (mmol/L) #	1.08(0.90-1.33)	  1.12(0.91-1.34)	  1.07(0.90-1.32)	0.022**	
hyperlipidaemia$	861(31.5%)	194(26.6%)	667(33.3%)	0.001**	
Cardiovascular disease $	417(12.6%)	 124(13.4%)	  293(12.3%)	0.405	
ACR (mg/g creatinine) #	342.25(73.64-897.93)	343.05(75.60-780.51)	341.72(72.56-936.66)	0.626	
ACR group $
1192
 601
 591
0.0102**

  1＜¼30mg/g
 198(16.61%)
 114(18.97%)
  84(14.21%)


  2=30-299mg/g
 400(33.56%)
 212(35.27%)
 188(31.81%)


  3≥300mg/g
 594(49.83%)
 275(45.76%)
 319(53.98%)


	3022			0.680	
	474（¨15.7%）©	127（¨15.6%）©	347（¨15.7%）©		
	955(31.6%)	248（¨30.5%）©	707（¨32.0%）©		
	1593(52.7%)	439（¨53.9%）©	1154（¨52.3%）©		
sCa mmol/L）©*	2.22(0.20)	2.22(0.18)	2.22(0.21)	0.145	
sP（¨mmol/L）©#	1.19(1.05-1.34)	1.17(1.05-1.31)	1.19(1.06-1.34)	0.018**	
CKD-MBD $	1707(57.0%)	467(54.6%)	 1240(58%)	0.083	
HCO3- （¨mmol/L）©*	25.26(4.08)	25.70(3.78)	 25.11 (4.16)	0.006**	
diabetes$	146(15.6)	200(23.8%)	582(27.8%)	0.028	
eGFR (ml/min/1.73m2) *	50.08(29.50)	57.22(32.68)	47.31(27.69)	<0.001**	
eGFR group $	938(100%)				
  ≥60 ml/min/1.73m2	360(38.4%)	139(26.7%)	221(52.9%)	<0.001**	
  30-60ml/min/1.73m2	345(36.8%)	212(40.8%)	133(31.8%)		
  15-30 ml/min/1.73m2	233(24.8%)	169(32.5%)	64(15.3%)		


Note 1: * The variable is numerical and statistics is Mean(Standard deviation), p-value calculated based on T test.
Note 2: # The variable is numerical and statistics is Median(Interquartile range), p-value calculated based on Wilcoxon test.
Note 3: $ The variable is character and statistics is Frequency(Percentage), p-value calculated based on Chi-square test.
Note 4: The denominator of Percentage is number of the variable.
Note 5: ** Statistically significant at 0.05.


Table S2. Relationship between hsCRP and ESRD, CVD and all-cause mortality events rates

hsCRP binary	Number of events	p for log-rank	
ESRD events 		0.506	
<3(N= 351)	57 (16.2%)		
≥3 (N=146 )	21(14.3 %)		
Total	78(15.7%)		
CVD events 		0.608	
<3 (N=351)	18(5.1%)		
≥3 (N=146 )	10(6.8%)		
Total	28(5.6%)		
All-cause mortality events 		0.368	
<3 (N=351)	16(4.6%)		
≥3 (N=146 )	10(6.8%)		
Total	26(5.2%)		
